# Comparison of Cold-Sprayed Coatings of Copper-Based Composite Deposited on AZ31B Magnesium Alloy and 6061 T6 Aluminum Alloy Substrates

**DOI:** 10.3390/ma16145120

**Published:** 2023-07-20

**Authors:** Na Xue, Weiwei Li, Ling Shao, Zhibiao Tu, Yingwei Chen, Sheng Dai, Nengyong Ye, Jitang Zhang, Qijie Liu, Jinfang Wang, Meng Zhang, Xinxing Shi, Tianle Wang, Mengliang Chen, Yingqi Huang, Feilong Xu, Liu Zhu

**Affiliations:** 1 Zhejiang Provincial Key Laboratory for Cutting Tools, Taizhou University, Taizhou 318000, China; xuena@tzc.edu.cn (N.X.); lww@tzc.edu.cn (W.L.); tutoptu@tzc.edu.cn (Z.T.); sdai@tzc.edu.cn (S.D.); yny1030@163.com (N.Y.); 18943979097@163.com (J.Z.); wjf0909@tzc.edu.cn (J.W.); cailiaoo@126.com (M.Z.); shixinxing@tzc.edu.cn (X.S.); wtl0203@tzc.edu.cn (T.W.); a731634915@hotmail.com (F.X.); 2 Taizhou Key Laboratory of Medical Devices and Advanced Materials, Research Institute of Zhejiang University-Taizhou, Taizhou 318000, China; cywsn@163.com (Y.C.); tanksman@163.com (Q.L.); yingqihuang77@hotmail.com (Y.H.); 3 School of Materials Science and Engineering, Zhejiang Sci-Tech University, Hangzhou 310018, China; mengliangchen2023@hotmail.com

**Keywords:** cold spray, surface roughness, thickness, microstructure, adhesion, particle velocity

## Abstract

Copper-coated graphite and copper mixture powders were deposited on AZ31B magnesium alloy and 6061 T6 aluminum alloy substrates under different process parameters by a solid-state cold spray technique. The microstructure of the copper-coated graphite and copper composite coatings was visually examined using photographs taken with an optical microscope and a scanning electron microscope. The surface roughness of the coatings was investigated with a 3D profilometer. The thickness of the coatings was determined through the analysis of the microstructure images, while the adhesion of the coatings was characterized using the scratch test method. The results indicate that the surface roughness of the coatings sprayed on the two different substrates gradually decreases as gas temperature and gas pressure increase. Additionally, the thickness and adhesion of the coatings deposited on the two different substrates both increase with an increase in gas temperature and gas pressure. Comparing the surface roughness, thickness, and adhesion of the coatings deposited on the two different substrates, the surface roughness and adhesion of the coatings on the soft substrate are greater than those of the coatings on the hard substrate, while the thickness of the coatings is not obviously affected by the hardness of the substrate. Furthermore, it is noteworthy that the surface roughness, thickness, and adhesion of the copper-coated graphite and copper composite coatings sprayed on the two different substrates exhibit a distinct linear relationship with particle velocity.

## 1. Introduction

Solid-state cold spray (CS) is a new technology that uses a supersonic jet of compressed gas to impact and bond the powder particles onto a substrate; meanwhile, the powder particles are heated by the jet of gas at a temperature below the melting point of the powder materials [1,2]. This enables the formation of coatings from solid-state particles, effectively reducing or eliminating detrimental effects such as evaporation, melting, high-temperature oxidation, undesired phase transformation, crystallization, grain growth, gas release, residual stresses, and other typical challenges encountered in conventional thermal spray technologies [3,4]. By eliminating the harmful impact of high temperatures on coatings and substrates, CS technology opens up remarkable opportunities and advantages, making it a highly promising technique for a wide range of industrial applications. Based on the above-mentioned points, the CS technique has received special attention as a promising candidate for a surface modification method of various materials as it generates a minimal heat-affected zone in both the as-deposited material and substrate [5]. Copper (Cu) [6], Cu-based alloys [7,8], Al [9], Al-based alloys [10,11], Ni [12], Ni-based alloys [13,14], Ti-based alloys [15,16], Fe-based alloys [8,17], Sn [18], high or medium entropy alloys [19,20], and metal-based composites such as Cu-Al_2_O_3_ [21], Al-SiC [22], Al-TiB_2_ [23], Al-Al_2_O_3_ [24], Ni-WC [25], etc., in the form of coatings or parts, have been successfully actualized by CS technology so far.

Light metal alloys (like magnesium alloy and aluminum alloy) play a crucial role in engineering industries, serving as key materials for the manufacturing of transportation equipment, aircraft components, aerospace systems, military hardware, and missile applications due to their low density, excellent stiffness, impressive strength-to-weight ratio, and favorable workability [26,27]. However, they have poor performance in wear resistance, corrosion resistance, low hardness, and ease of deformation. Surface treatment techniques are necessary to be implemented for improving the surface properties, such as surface hardness, wear resistance, and corrosion resistance, of magnesium alloy, aluminum alloy, and other light metal alloys. Copper-coated graphite and copper composites (Cu@Gr/CC) provide a solution to the issue of inadequate wetting between graphite and Cu, and successfully integrate the favorable characteristics of graphite and Cu, leading to a low friction coefficient, good thermal conductivity, and impressive electrical conductivity [28,29]. Consequently, Cu@Gr/CC coatings are highly appropriate for applying onto the surfaces of magnesium alloy and aluminum alloy, effectively enhancing their electrical and mechanical properties.

The deposition of ductile and soft materials on a soft substrate (soft-on-soft interface) by the CS technique is the most effective, compared to soft-on-hard, hard-on-soft, and hard-on-hard interfaces [30]. In this study, Cu-coated graphite (Cu@graphite) and Cu mixture powders were deposited on magnesium alloy and aluminum alloy by the CS technique. Single-pass and single-layer Cu@Gr/CC coatings sprayed on different substrates were made. The cold spray ability metrics (surface roughness *R*a, thickness, and adhesion) of the Cu@Gr/CC coatings deposited on magnesium alloy and aluminum alloy were characterized with a 3D profilometer, an optical microscope (OM), and a scratch tester, respectively, and compared with each other. The appearance, microstructure, and element constitution were examined by an OM and a scanning electron microscope (SEM). And the relationships of particle velocity (*V*_p_) and *R*a, thickness, microstructure, and adhesion were analyzed. This work provides the data reference for multi-pass and multi-layer Cu@Gr/CC coatings deposited on different substrates in future work.

## 2. Experimental

### 2.1. Feedstock Materials and Substrates

AZ31B magnesium alloy and 6061 T6 aluminum alloy plates, both measuring 100 mm × 100 mm × 3 mm, were employed as the substrate materials. The nominal chemical compositions of the alloys are provided in Table 1. Prior to the deposition process, the surfaces of all substrates were prepared by grinding using an SJK 9070F dry sand blasting machine (Changzhou Instant Clean Machine Technology Co., Ltd., Changzhou, Jiangsu, China) and subsequently cleaned in an ultrasonic cleaner with alcohol. The surface roughness of the AZ31B magnesium alloy and 6061 T6 aluminum alloy plates after sandblasting is 3.82 μm and 3.97 μm, respectively. A water-atomized Cu powder (99.99% purity) with a volume-weighted mean particle size of 17.4 μm was utilized. Additionally, an electroplated Cu@graphite powder (30 wt.% graphite) with a volume-weighted average particle size of 18.6 μm was employed as a reinforcement. A mixture of Cu@graphite and Cu powders, comprising 7 wt.% Cu@graphite powders, was milled in an MITR QM-QX-4L 360° omnidirectional planetary ball mill for 1 h with 200 rpm rotation speed and 1 h rotation duration using a PTFE container (Changsha Miqi Equipment Co., Ltd., Changsha, Hunan, China) and agate balls to ensure homogeneity. The microstructure and morphology of the powder mixture were characterized by a Hitachi S-4800 scanning electron microscope (Hitachi, Tokyo, Japan).

### 2.2. Deposit Preparation

**Figure 1:**
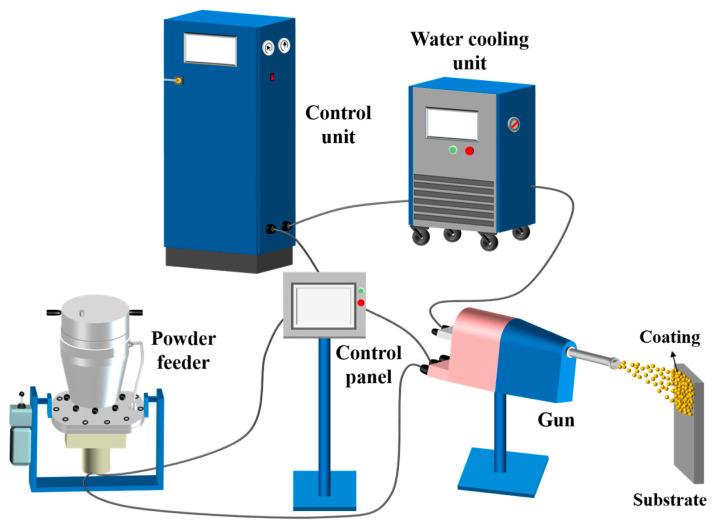
Diagram of the operating principle of cold spray process.

To achieve high-quality Cu@Gr/CC coatings on AZ31B magnesium alloy and 6061 T6 aluminum alloy substrates, it was essential to conduct an initial investigation focusing on single-pass and single-layer coatings. CS PCS-100 equipment (Plasma Giken Co., Ltd., Osato, Saitama, Japan) was used to deposit Cu@graphite and Cu mixture powders on grit blasted two different substrates. Single-pass and single-layer Cu@Gr/CC coatings were produced at different spray deposition parameters. The commercial CS PCS-100 system was equipped with a PNFC-010 convergent-divergent (de-Laval type) nozzle with a round shape exit, and N_2_ was employed as a carrier gas and process gas, as illustrated in Figure 1. The spray gun was moved across the substrate at a constant linear speed of 50 mm s^−1^ while maintaining a fixed stand-off distance of 20 mm. The gas flow rate during the deposition process was set at a consistent value of 180 standard liters per minute (SLM) for all powder blends. The gas pressure was adjusted between 4 and 5.5 MPa, and gas temperature was varied in the range of 600 to 900 °C at each gas pressure. The specific process parameters used in this study are provided in Table 2.

### 2.3. Deposit Characterization

To examine the microstructure and measure the thickness of the single-pass and single-layer Cu@Gr/CC coatings, the samples were wire-cut into dimensions of 10 mm × 10 mm × 3 mm along the cross-sections. The cross-sectional surfaces of the specimens were subjected to wet grinding using abrasive papers, followed by mechanical polishing to achieve mirror-polished sections. Subsequently, the specimens underwent ultrasonic cleaning in alcohol. The cross sections of the samples were eroded with a mixture solution of 5 g FeCl_3_, 10 mL HCl, and 100 mL H_2_O for a better observation of the microstructure of the cold-sprayed coatings. The cross-sectional deposit microstructure and element constitution of the deposited coatings were investigated using a Zeiss Axio scope A1 optical microscope (Carl Zeiss, Jena, Thuringia, Germany) and an SEM furnished with an energy dispersive spectroscope (EDS) (Thermo Fisher Scientific, Waltham, MA, USA). The thickness of the coatings was assessed at the highest point using an OM. The *R*a (according to ISO 4287 standard [31]) of the deposited coatings was evaluated using a Bruker Contour X100 3D profilometer (Bruker, Billerica, MA, USA) with a maximum measuring range of 500 μm and a resolution of 0.1 nm. The measurements were conducted with the assistance of Bruker Vision64 software, and the reported *R*a values represent the average of ten readings.

### 2.4. Adhesion Test

**Figure 2:**
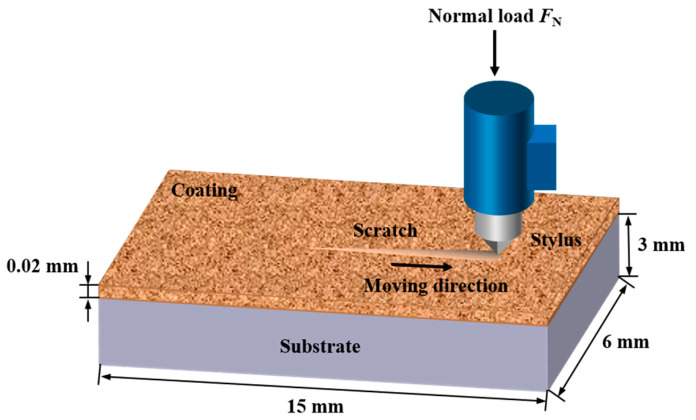
Schematic diagram of the scratch adhesion test for the coatings.

The adhesive properties of Cu@Gr/CC coatings sprayed on AZ31B magnesium alloy and 6061 T6 aluminum alloy substrates were evaluated utilizing a WS-2005 scratch tester (Lanzhou Zhongke Kaihua Technology Development Co., Ltd., Lanzhou, Gansu, China). Scratches were generated on the coated sample using a three-sided pyramidal diamond (Berkovich) indenter with a tip radius of 0.2 mm. The scratches were drawn across the specimen surface under a progressively increasing load of 100 N min^−1^, a total load of 100 N, and a scratch length of 4 mm. Shedding or delamination damage of the cold-sprayed coating occurs along the scratch path with adhesive failure indicating the separation of the coating from the substrate. Figure 2 presents the schematic diagram of scratch coating adhesion test. After grinding and measuring, the thickness of the coating was controlled at 20 μm and the surface roughness of the coating reached 0.25 μm. Before testing, the coating surface was polished to achieve a mirror finish. The scratch test was performed three times for each specimen, and the average value was considered as the measure of adhesion for the deposited coatings. The adhesion of the deposited coatings on the substrates was characterized by examining the scratch morphology using SEM and determining the critical load. The adhesion of the coatings was evaluated based on the position of coating delamination (the critical length of the scratch, *L*_c_) within the total length of the scratch (*L*_total_) with 4 mm, under a total load of 100 N. The adhesion of the coatings was determined according to the following equation as established in [32]:

(1) $$\mathrm{Adhesion}=\frac{L_{c}}{L_{\mathrm{total}}}\times 100$$

## 3. Results

### 3.1. Microstructure, Surface Roughness, and Thickness

**Figure 3:**
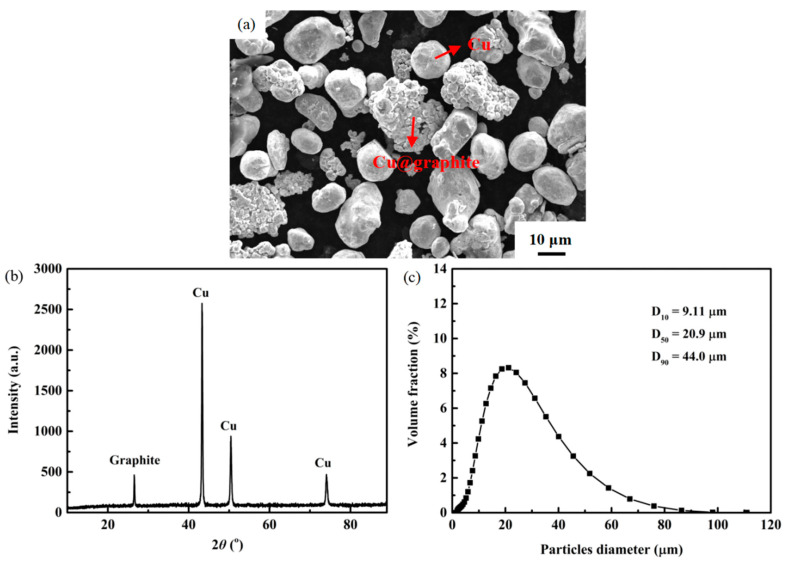
SEM photograph illustrating the morphology of the mixture particles containing 7 wt.% Cu-coated graphite and Cu (**a**), XRD pattern revealing the phases present in the mixed powders (**b**) and the size distribution curve of the mixed particles (**c**).

An SEM photo of the microstructure and morphology of the powder mixture used for cold spray is presented in Figure 3a. It is observed that Cu@graphite particles possess a core–shell structure, while Cu particles exhibit a near-spherical surface structure. The powder mixtures used as feedstock materials were inspected using a Bruker D8 Advance X-ray diffractometer (XRD) with a scanning speed of 3° per minute, scanning step of 0.02°, 2*θ* range of 10–90°, and Cu K_α_ radiation (Figure 3b). From Figure 3b, it can be known that the powder mixtures are composed of graphite-3R and Cu phases. The lattice parameters of the graphite-3R phase are a = 2.456 nm, b = 2.456 nm, c = 10.044 nm, α = 90°, β = 90°, γ = 120°, and V = 52.5 nm^3^ (from PDF Card No. 26-1079). The lattice parameters of the Cu phase are a = 3.615 nm, b = 3.615 nm, c = 3.615 nm, α = 90°, β = 90°, γ = 90°, and V = 47.2 nm^3^ (from PDF Card No. 04-0836). The powder size distribution of the mixed powders was measured using a Mastersizer3000 laser diffraction particle size analyzer (Malvern Instruments Ltd., Malvern, UK), yielding D_10_ of 9.11 μm, D_50_ of 20.9 μm, and D_90_ of 44.0 μm (where D_10_, D_50_, and D_90_ indicate that 10, 50, and 90% of the particles are smaller than this diameter, respectively), as shown in Figure 3c.

**Figure 4:**
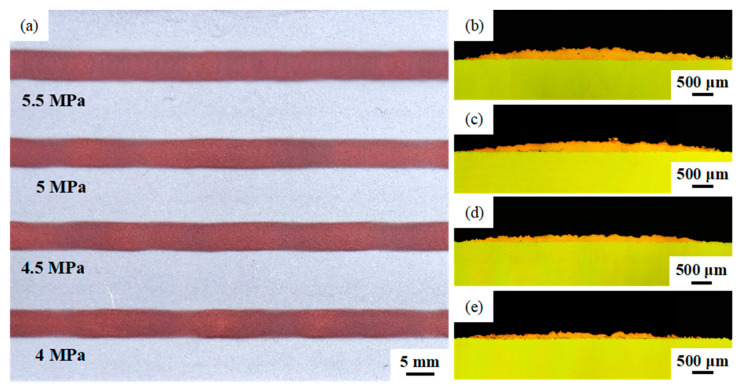
Visual characteristics of the single-pass and single-layer Cu@Gr/CC coatings deposited on 6061 T6 aluminum alloy substrate at 800 °C and different gas pressures (**a**), and cross-sectional morphologies of these cold-sprayed coatings: 5.5 MPa (**b**), 5 MPa (**c**), 4.5 MPa (**d**), and 4 MPa (**e**).

The visual characteristics of the deposited coatings of the single-pass and single-layer Cu@Gr/CC at 800 °C under various gas pressures sprayed on the 6061 T6 aluminum alloy substrate of 100 mm × 100 mm × 3 mm were examined, as shown in Figure 4a. Figure 4a is the view from the top of the coatings. The cold-sprayed coatings are undulating and uneven, and the width of the coatings is about 6 mm. The coatings made at other temperatures for the 6061 T6 aluminum alloy substrate or deposited on the AZ31B magnesium alloy substrate have a similar appearance. Figure 4b–e show the cross-sectional OM images of the Cu@Gr/CC coatings sprayed on the 6061 T6 aluminum alloy substrate at 800 °C and various gas pressures. The sprayed coatings can be observed on the pale green 6061 T6 aluminum alloy substrate. The interfaces between the coatings and substrates are all obvious, and the coatings are a fan-shaped deposition on the substrates.

**Figure 5:**
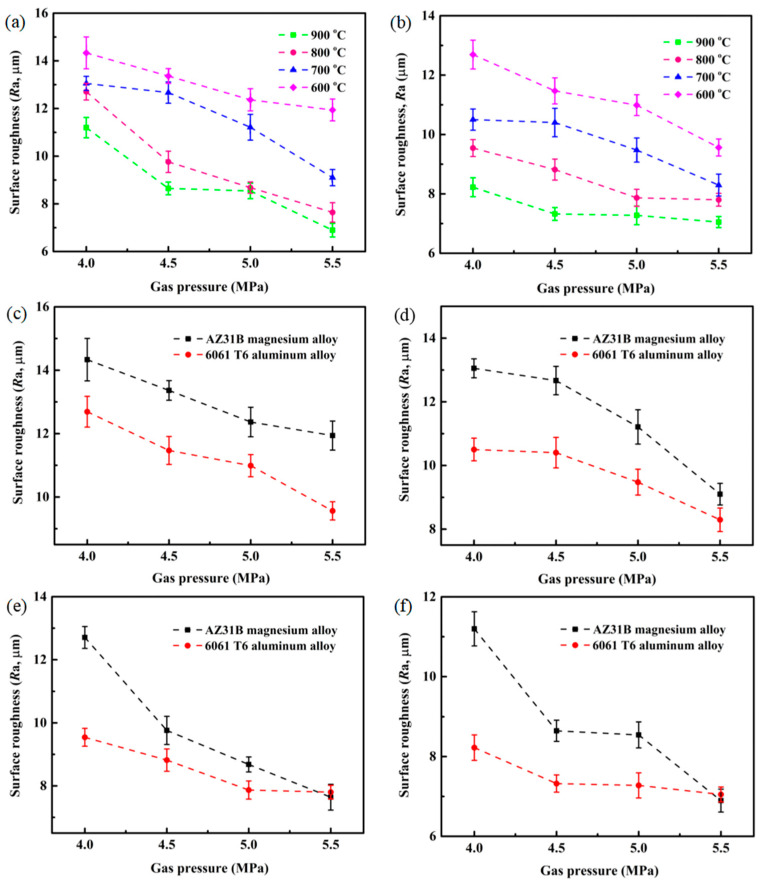
*R*a of the sprayed Cu@Gr/CC coatings on different substrates under different cold spray parameters: AZ31B magnesium alloy (**a**), 6061 T6 aluminum alloy (**b**), 600 °C (**c**), 700 °C (**d**), 800 °C (**e**), and 900 °C (**f**).

Similar to Cu@Gr/CC coatings sprayed on the 6061 T6 aluminum alloy substrate (Figure 5b), the Cu@Gr/CC coatings sprayed on the AZ31B magnesium alloy substrate demonstrate a comparatively rough surface (Figure 5a). The *R*a of the single-pass and single-layer Cu@Gr/CC coatings sprayed on the 6061 T6 aluminum alloy substrate ranges from 7.05 to 12.69 μm, while on AZ31B magnesium alloy substrate, the *R*a of the coatings ranges from 6.90 to 14.44 μm. The *R*a of the coatings sprayed on different substrates (AZ31B magnesium alloy and 6061 T6 aluminum alloy) gradually decreases with an increase in gas temperature and gas pressure. From Figure 5c–f, it can be obviously seen that the *R*a of the coatings deposited on the AZ31B magnesium alloy substrate is higher than that of the coatings deposited on the 6061 T6 aluminum alloy substrate under the same cold spray parameters. This illustrates that the Cu@Gr/CC coatings have a lower *R*a for deposition on the hard substrate than on the soft substrate by the CS technology.

**Figure 6:**
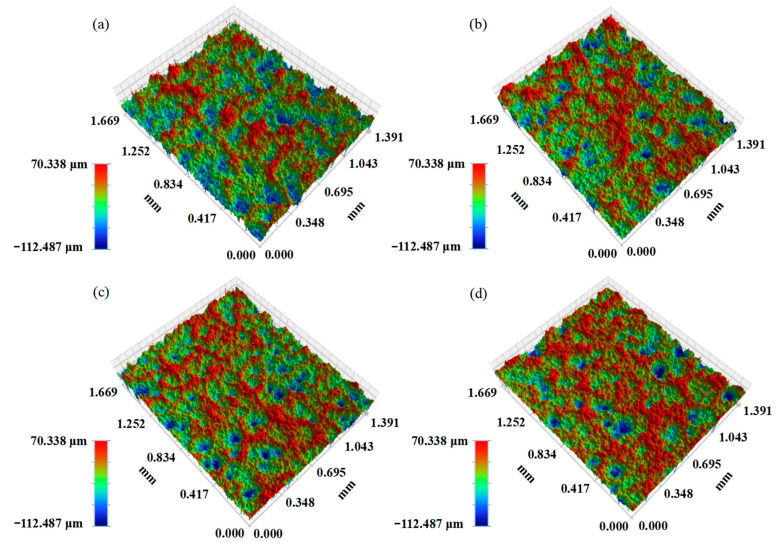
Three-dimensional surface morphology of the Cu@Gr/CC coatings deposited on AZ31B magnesium alloy substrate at 600 °C under various gas pressures: 4 MPa (**a**), 4.5 MPa (**b**), 5 MPa (**c**), and 5.5 MPa (**d**).

Figure 6 illustrates the top surface morphology and 3D profile of the single-pass and single-layer Cu@Gr/CC coatings sprayed on the AZ31B magnesium alloy substrate at 600 °C under various gas pressures. Bulges and depressions are observed on the 3D surface morphology of the deposited coatings, and the bulges and depressions are less and less prominent with increased gas pressure. Because of the spreading effect, wave lines are formed on the facets vertical to the deposition direction. Some spread powder mixtures were subtracted and cavities remained on the coating surfaces.

**Figure 7:**
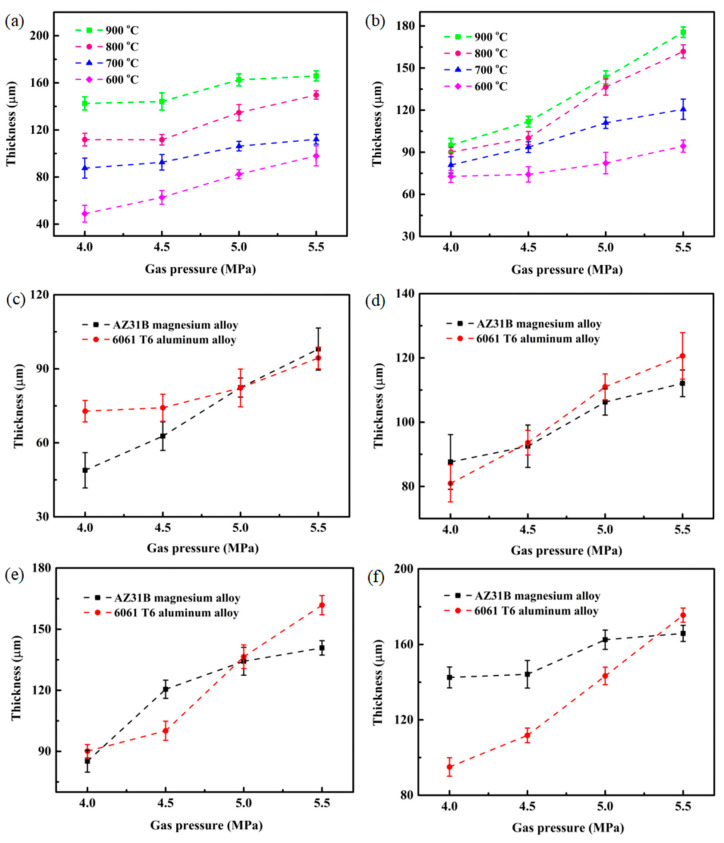
Impact of spraying parameters on the thickness of the Cu@Gr/CC coatings deposited on different substrates: AZ31B magnesium alloy (**a**), 6061 T6 aluminum alloy (**b**), 600 °C (**c**), 700 °C (**d**), 800 °C (**e**), and 900 °C (**f**).

The influence of spraying parameters on the thickness of the Cu@Gr/CC coatings deposited on AZ31B magnesium alloy and 6061 T6 aluminum alloy substrates is shown in Figure 7. The thickness of the coatings deposited on both substrates increases noticeably with higher gas temperature and gas pressure (Figure 7a,b). This phenomenon can be attributed to the fact that higher cold spray parameters (gas temperature and gas pressure) result in increased deceleration of powder particles in the stagnation zone [33]. The coatings sprayed on both substrates at 900 °C and 5.5 MPa exhibit the highest thickness, measuring 165.85 μm and 175.56 μm, respectively. Conversely, the coatings deposited on both substrates at 600 °C and 4 MPa demonstrate the lowest thickness, measuring 48.86 μm and 72.82 μm, respectively. To compare the thickness of the Cu@Gr/CC coatings sprayed on both substrates, no clear trend emerges regarding which substrate consistently exhibits higher thickness at the same cold spray parameters (Figure 7c–f).

**Figure 8:**
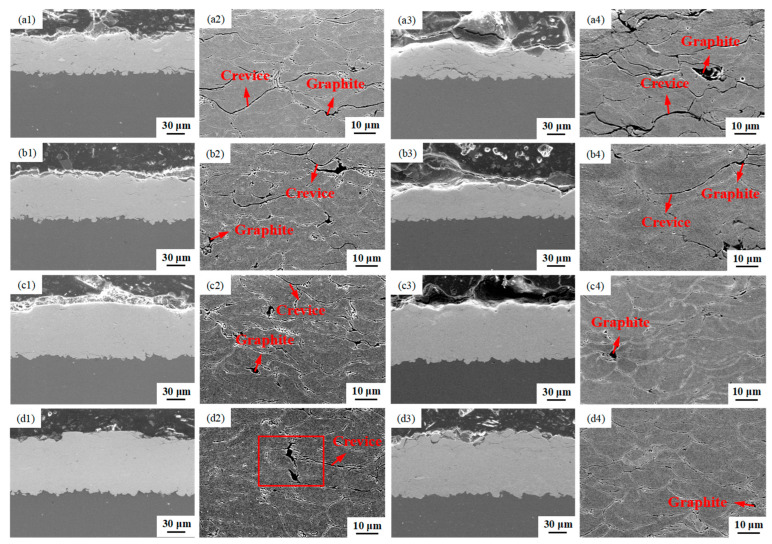
SEM photographs illustrating the thickness and microstructure of the Cu@Gr/CC coatings deposited on different substrates at 800 °C under various gas pressures. AZ31B magnesium alloy substrate: 4 MPa (**a1**,**a2**), 4.5 MPa (**b1**,**b2**), 5 MPa (**c1**,**c2**), and 5.5 MPa (**d1**,**d2**); 6061 T6 aluminum alloy substrate: 4 MPa (**a3**,**a4**), 4.5 MPa (**b3**,**b4**), 5 MPa (**c3**,**c4**), and 5.5 MPa (**d3**,**d4**).

Figure 8 displays SEM photographs of the thickness and microstructure of the corroded specimens of the Cu@Gr/CC coatings spayed on AZ31B magnesium alloy and 6061 T6 aluminum alloy substrates at 800 °C under various gas pressures. In Figure 8(a1,b1,c1,d1) (cross-sectional SEM micrographs of the coatings deposited on as-received AZ31B magnesium alloy substrate) and Figure 8(a3,b3,c3,d3) (cross-sectional SEM images of the coatings sprayed on the as-received 6061 T6 aluminum alloy substrate), only a limited number of porosities are observed within the cross-sections of the deposited coatings. The observation clearly shows that the thickness of the sprayed coatings increases as the gas pressure increases for both the coatings deposited on soft and hard substrates. The microstructures of the coatings sprayed at various gas pressures and different substrates are further revealed in higher magnification. The compaction of the coatings deposited on the AZ31B magnesium alloy substrate increases with the increase in the working pressure of the cold spray process (Figure 8(a2,b2,c2,d2)). A similar trend is shown in the compaction of the coatings deposited on the 6061 T6 aluminum alloy substrate (Figure 8(a4,b4,c4,d4)). Owing to the higher gas pressure, more plastic deformation of the powder particles occurs during the CS process. Crevices are observed next to loosely adhered particles in the Cu@Gr/CC coatings sprayed on the soft and hard substrates at 800 °C and 4 MPa, as well as 800 °C and 4.5 MPa (Figure 8(a2,b2,a4,b4)). Compared with the coatings deposited on the AZ31B magnesium alloy substrate at 800 °C and 5 MPa, and 800 °C and 5.5 MPa (Figure 8(c2,d2)), the coatings deposited on the 6061 T6 aluminum alloy substrate at the same cold spray parameters are denser (Figure 8(c4,d4)). It is evident that whether spraying on soft substrate or on hard substrate, both the deposited coatings become more and more dense with increased *V*_p_.

**Figure 9:**
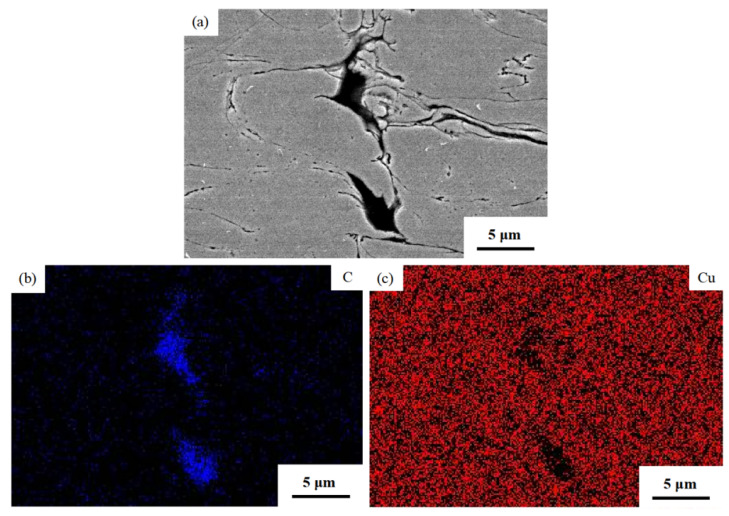
Element scanning maps of a specific region indicated using a rectangular box within the Cu@Gr/CC coating sprayed on AZ31B magnesium alloy substrate at 5.5 MPa and 800 °C (as shown in Figure 8(d2)): SEM photograph (**a**), C (**b**), and Cu (**c**).

Figure 9 depicts the element scanning maps of a specific region highlighted by a rectangular box within the Cu@Gr/CC coating deposited on the AZ31B magnesium alloy substrate at 800 °C and 5.5 MPa (Figure 8(d2)). The EDS mapping reveals that the black area consists of carbon (C) elements, while the gray region consists of Cu elements. This indicates that the black region corresponds to deformed graphite. Due to weak deformation, Cu@graphite particles almost maintain a granular shape.

### 3.2. Adhesion

**Figure 10:**
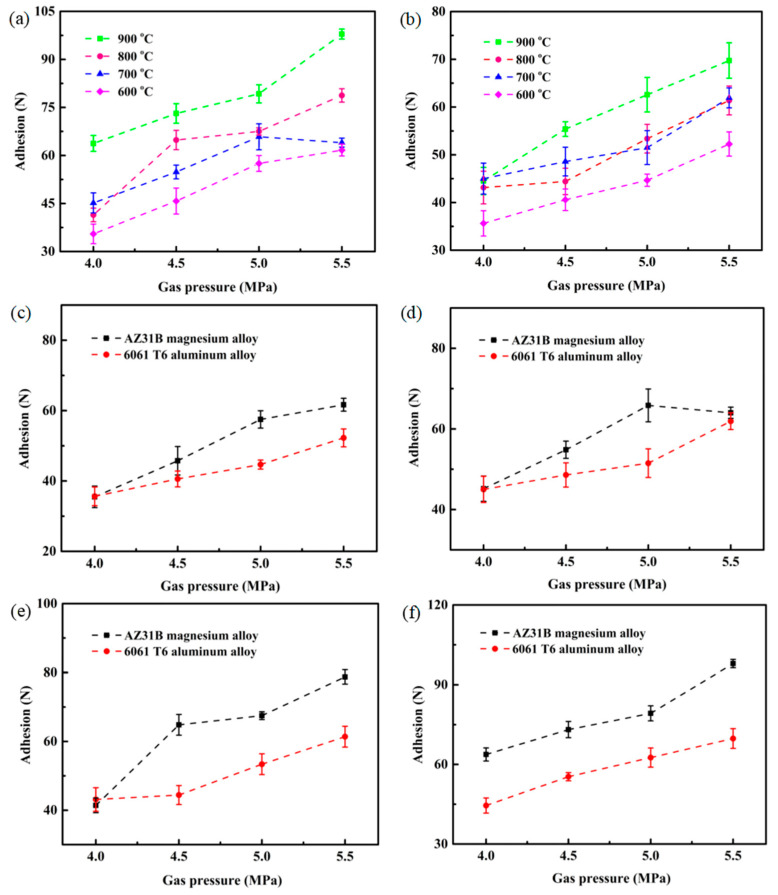
Adhesion of the Cu@Gr/CC coatings on different substrates under different deposited parameters: AZ31B magnesium alloy (**a**), 6061 T6 aluminum alloy (**b**), 600 °C (**c**), 700 °C (**d**), 800 °C (**e**), and 900 °C (**f**).

The adhesion of the Cu@Gr/CC coatings sprayed on different substrates under different spraying parameters is shown in Figure 10. Regardless of whether the coatings are deposited on the AZ31B magnesium alloy substrate or 6061 T6 aluminum alloy substrate, the adhesion of the coatings increases as gas pressure increases. Additionally, the adhesion of the coatings also increases as gas temperature increases, although the effect is not as pronounced as that of gas pressure (Figure 10a,b). The highest average adhesion value at the interface between the coating and the substrate is 97.92 N for the coatings sprayed on the AZ31B magnesium alloy substrate and 69.75 N for the coatings deposited on the 6061 T6 aluminum alloy substrate, at 900 °C and 5.5 MPa. The lowest average adhesion value at the interface between the coating and the substrate is 35.50 N for the coatings sprayed on the AZ31B magnesium alloy substrate and 35.63 N for the coatings sprayed on the 6061 T6 aluminum alloy substrate, at 600 °C and 4 MPa. From Figure 10c–f, it can be obviously seen that the adhesion of the coatings sprayed on the AZ31B magnesium alloy substrate is close to that of the coatings deposited on the 6061 T6 aluminum alloy substrate at 600 °C and 4 MPa, 700 °C and 4 MPa, and 800 °C and 4 MPa. As the gas pressure increases, the adhesion of the coatings deposited on the AZ31B magnesium alloy substrate is higher than that of the coatings deposited on the 6061 T6 aluminum alloy substrate at 600 °C, 700 °C, and 800 °C. However, the adhesion of the coatings sprayed on the AZ31B magnesium alloy substrate is entirely higher than that of the coatings deposited on the 6061 T6 aluminum alloy substrate at 900 °C and different gas pressures. This indicates that the interfacial bonding strength of the coatings is mainly influenced by the substrate hardness [34].

**Figure 11:**
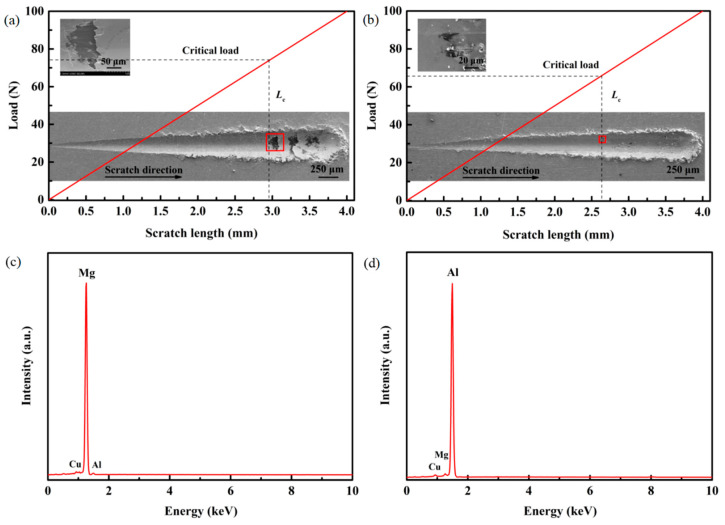
SEM photographs of *L*_c_ after scratch testing and the corresponding critical load for the Cu@Gr/CC coatings deposited on different substrates at 800 °C and 5.5 MPa: AZ31B magnesium alloy (**a**) and 6061 T6 aluminum alloy (**b**). EDS results of the delaminated regions of the Cu@Gr/CC coatings sprayed on different substrates at 800 °C and 5.5 MP: AZ31B magnesium alloy (**c**) and 6061 T6 aluminum alloy (**d**). The shed coating positions are noted by rectangular boxes in the scratches.

The typical SEM images of scratches of the Cu@Gr/CC coatings deposited on the two different substrates at 800 °C and 5.5 MPa are illustrated in Figure 11a,b, respectively. The light area represents the length of the damaged region caused by the diamond indenter. Comparing the SEM photographs of the *L*_c_ after scratch testing, it can be observed that the *L*_c_ and the corresponding critical load are higher for the coating deposited on the AZ31B magnesium alloy substrate compared to the coating on the 6061 T6 aluminum alloy substrate at 800 °C and 5.5 MPa. When the applied load reaches the critical load, the shedding of the coating from the substrate occurs along the direction of the scratch. The shed coating positions, highlighted in Figure 11a,b, are magnified and shown in the top left corner, respectively. Figure 11c,d present EDS results of the shed coating positions noted by rectangular boxes in Figure 11a,b. Mg and Al elements are both detected in the delaminated regions of the coatings deposited on the two different substrates.

**Figure 12:**
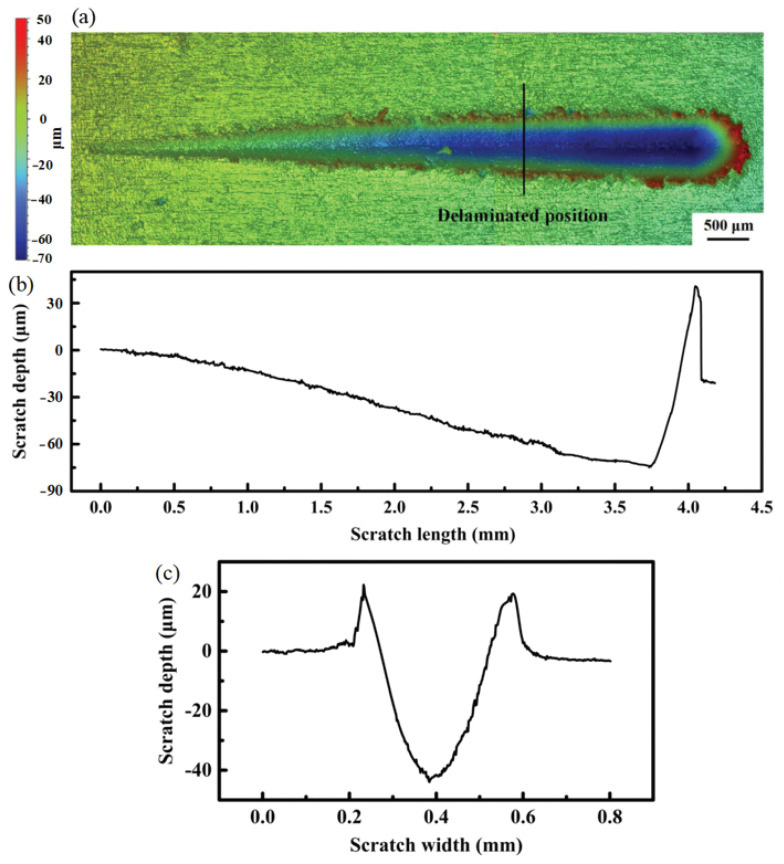
Results of scratch test of the coating deposited on 6061 T6 aluminum alloy substrate at 800 °C and 5.5 MPa: 3D surface morphology of the scratch groove (**a**), the relation of scratch depth and scratch length (**b**), and the groove depth of the delaminated position (**c**).

The profile of the typical scratch formed after the scratch test was characterized with a 3D profilometer. The analyzed results of the scratch of the coating sprayed on the 6061 T6 aluminum alloy substrate at 800 °C and 5.5 MPa are presented in Figure 12. Figure 12a illustrates the 3D surface morphology of the scratch groove formed by the diamond indenter as the applied load is increased linearly from 0 to 100 N. It can be observed that the groove width increases with an increase in applied load. The depth of the scratch groove gradually increases from the starting point to the end of the scratch on the deposited coating (Figure 12b). The groove depth at the delaminated position, as shown in Figure 12a, reaches 43 μm (Figure 12c).

## 4. Discussion

*V*_p_ plays a vital role in the cold spray process as it directly influences the effectiveness and the bonding strength of jet formation for the deposited coatings [35]. Champagne et al. [36] proposed an empirical and simple approach to evaluate the *V*_c_ needed for the achievement of interfacial mixing by the following equation,

(2) $$V_{c}={\left[\left(7.5\times 10^{4})(\frac{B}{\rho }\right)\right]}^{0.5}$$

where *V*_c_ is the critical *V*_p_ in meters per second, *B* corresponds to the substrate Brinell hardness, and *ρ* denotes the particle density given in kilograms per cubic meter. Meanwhile, Champagne et al. calculated the *V*_c_ of 500 m s^−1^ with Equation (2) for Cu particles impacting 6061 T6 aluminum alloy substrate. The Brinell hardness of 6061 T6 aluminum alloy is twice that of AZ31B magnesium alloy, with values of 90 and 45, respectively. The *V*_c_ with Equation (2) for Cu particles impacting the AZ31B magnesium alloy substrate is 354 m s^−1^. Therefore, during the spray deposition process, the *V*_p_ of Cu particles on the 6061 T6 aluminum alloy substrate should be greater than 500 m s^−1^, and the *V*_p_ of Cu particles on the AZ31B magnesium alloy substrate should be greater than 354 m s^−1^. To determine the *V*_p_, an empirical equation is employed as shown below [37,38],

(3) $$V_{p}=\frac{V_{g}}{1+0.85\sqrt{\frac{D}{x}}\sqrt{\frac{\rho_{p}V_{g}^{2}}{p_{o}}}}$$

where *V*_g_ represents the gas velocity, *D* is the particle diameter, *x* denotes the axial position of 20 mm, *ρ*_p_ is the particle density of 2.35 × 10^3^ kg m^−3^, and *p*_o_ corresponds to the gas supply pressure measured at the nozzle inlet. The *V*_g_ is given by [39],

(4) $$V_{g}=M\sqrt{\frac{\gamma RT}{M_{w}}}$$

where M is the local Mach number of 1.35, γ represents the ratio of specific heats of 1.4, *T* denotes the gas temperature, and *M*_W_ stands for the molecular weight of the gas. Equation (3) can be rewritten as

(5) $$V_{p}=\frac{M\sqrt{\frac{\gamma RT}{M_{W}}}}{1+0.85M\sqrt{\frac{D}{x}}\sqrt{\frac{\rho_{p}\gamma RT}{p_{o}M_{W}}}}$$

According to Equation (5), cold spray parameters (gas temperature and gas pressure) in Table 2 are determined, with the result that *V*_p_ can be higher than the *V*_c_ of 500 m s^−1^ for Cu particles impacting the 6061 T6 aluminum alloy substrate during the spray deposition process, and *V*_p_ can be higher than the *V*_c_ of 354 m s^−1^ for Cu particles impacting the AZ31B magnesium alloy substrate during the cold spray process. The cold spray parameters (gas temperature and gas pressure) in Table 2 substitute Equation (5) to obtain the *V*_p_ under different spray deposition parameters. Table 3 shows the *V*_p_ at different cold spray parameters. It is observed from Table 3 that *V*_p_ exhibits an increase with higher working temperature and pressure of the spray deposition process gas. This phenomenon can be attributed to higher *V*_g_, leading to increased accelerating drag and elevated gas temperature, resulting in an increase in *V*_p_ [40].

**Figure 13:**
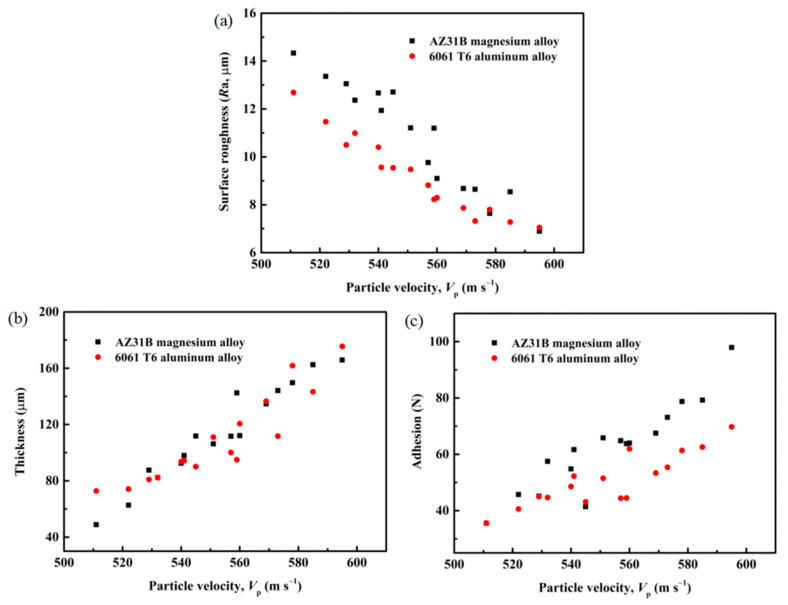
*R*a, thickness, and adhesion as a function of *V*_p_ for the cold-sprayed coatings of the single-pass and single-layer Cu@Gr/CC at various gas temperatures and gas pressures deposited on different substrates: correlation between *R*a and *V*_p_ (**a**); correlation between thickness and *V*_p_ (**b**); and correlation between adhesion and *V*_p_ (**c**).

The *R*a, thickness, and adhesion of the Cu@Gr/CC coatings sprayed on both AZ31B magnesium alloy and the 6061 T6 aluminum alloy substrates all exhibit a linear relationship with *V*_p_, as depicted in Figure 13. For both soft and hard substrates, the *R*a of the coatings demonstrates a linear decrease as *V*_p_ increases (Figure 13a). The primary factor contributing to the reduced *R*a is likely the reduced taper caused by the higher particle velocity and specific dose, rather than the impact velocity itself [41]. The thickness and adhesion of the Cu@Gr/CC coatings sprayed on both the soft and hard substrates reveal a linear increase as *V*_p_ increases, as shown in Figure 13b,c, respectively. Higher *V*_p_ can provide greater kinetic energy, causing the particles to generate higher impact force when they collide with the substrate surface. This impact force can induce plastic deformation and higher penetration depth of the particles on the substrate, resulting in a thicker coating and a higher adhesion between the coating and substrate [42].

## 5. Conclusions

This study investigates the deposition behavior of Cu@graphite and Cu mixture powders in the CS process, as well as the *R*a, thickness, microstructure, and adhesion of the Cu@Gr/CC coatings deposited on AZ31B magnesium alloy and 6061 T6 aluminum alloy substrates under different spraying parameters. These findings reveal that the hardness of the substrate significantly influences the *R*a and adhesion of the Cu@Gr/CC coatings, but not their thickness. Specifically, the coatings deposited on the AZ31B magnesium alloy substrate exhibit higher *R*a values compared to those on the 6061 T6 aluminum alloy substrate under the same cold spray parameters. The trend of coating thickness between soft and hard substrates at the same spraying parameters is not significant. Furthermore, the adhesion of the coatings sprayed on the AZ31B magnesium alloy substrate is generally higher than that on the 6061 T6 aluminum alloy substrate under the same spraying parameters. The *R*a of the coatings sprayed on both soft and hard substrates shows a linear decrease relationship with *V*_p_, while the thickness and adhesion of the coatings sprayed on both substrates have a linear increase relationship with *V*_p_. Additionally, the deposited coatings become denser with increased *V*_p_, regardless of the substrate hardness.

For studying the bonding mechanism between coating and substrate, the isolated Cu particle splat experiment and the influence of surface roughness of the substrate on the quality of Cu@Gr/CC coatings should be conducted. Based on the data of single-pass and single-layer Cu@Gr/CC coatings deposited on different substrates, multi-pass and multi-layer Cu@Gr/CC coatings deposited on different substrates will be performed. Future research will involve the examination of electrical conductivity, wear resistance, and corrosion resistance tests to investigate the usefulness of Cu@Gr/CC coatings.

## Figures and Tables

**Table 1:** Nominal chemical compositions (wt.%) of AZ31B magnesium alloy plate and 6061 T6 aluminum alloy plate.

Elements	Si	Fe	Cu	Cr	Mn	Ti	Zn	Ni	Al	Mg
AZ31B magnesium alloy plate	0.16	0.003	0.006	—	0.32	—	0.61	0.001	3.05	Balance
6061 T6 aluminum alloy plate	0.4–0.8	0–0.7	0.15–0.4	0.04–0.35	0.15 max	0.15 max	0.25 max	—	Balance	0.8–1.2

**Table 2:** Process parameters of Cu@Gr/CC coatings employed by CS technology.

Coating	Substrate	Gas Temperature (°C)	Gas Pressure (MPa)	Gas Flow of Feeding (SLM)	Traverse Velocity (mm s^−1^)	Distance from the Substrate (mm)	Deposition Layer (Layer)
1	AZ31B	900	5.5	180	50	20	1
2	AZ31B	900	5	180	50	20	1
3	AZ31B	900	4.5	180	50	20	1
4	AZ31B	900	4	180	50	20	1
5	AZ31B	800	5.5	180	50	20	1
6	AZ31B	800	5	180	50	20	1
7	AZ31B	800	4.5	180	50	20	1
8	AZ31B	800	4	180	50	20	1
9	AZ31B	700	5.5	180	50	20	1
10	AZ31B	700	5	180	50	20	1
11	AZ31B	700	4.5	180	50	20	1
12	AZ31B	700	4	180	50	20	1
13	AZ31B	600	5.5	180	50	20	1
14	AZ31B	600	5	180	50	20	1
15	AZ31B	600	4.5	180	50	20	1
16	AZ31B	600	4	180	50	20	1
17	6061 T6	900	5.5	180	50	20	1
18	6061 T6	900	5	180	50	20	1
19	6061 T6	900	4.5	180	50	20	1
20	6061 T6	900	4	180	50	20	1
21	6061 T6	800	5.5	180	50	20	1
22	6061 T6	800	5	180	50	20	1
23	6061 T6	800	4.5	180	50	20	1
24	6061 T6	800	4	180	50	20	1
25	6061 T6	700	5.5	180	50	20	1
26	6061 T6	700	5	180	50	20	1
27	6061 T6	700	4.5	180	50	20	1
28	6061 T6	700	4	180	50	20	1
29	6061 T6	600	5.5	180	50	20	1
30	6061 T6	600	5	180	50	20	1
31	6061 T6	600	4.5	180	50	20	1
32	6061 T6	600	4	180	50	20	1

**Table 3:** Particle velocity *V*_p_ at different cold spray parameters.

Sample	1, 17	2, 18	3, 19	4, 20	5, 21	6, 22	7, 23	8, 24	9, 25	10, 26	11, 27	12, 28	13, 29	14, 30	15, 31	16, 32
*V*_p_ (m s^−1^)	595	585	573	559	578	569	557	545	560	551	540	529	541	532	522	511

## Data Availability

The authors confirm that the data supporting the findings of this study are available within the article.
